# Nosocomial Severe Fever with Thrombocytopenia Syndrome in Companion Animals, Japan, 2022

**DOI:** 10.3201/eid2903.220720

**Published:** 2023-03

**Authors:** Hirohisa Mekata, Kazumi Umeki, Kentaro Yamada, Kunihiko Umekita, Tamaki Okabayashi

**Affiliations:** University of Miyazaki, Miyazaki, Japan (H. Mekata, K. Yamada, K. Umekita, T. Okabayashi);; Kyushu University of Health and Welfare, Nobeoka, Japan (K. Umeki);; University of Miyazaki Hospital, Miyazaki (K. Umekita)

**Keywords:** severe fever with thrombocytopenia syndrome, severe fever with thrombocytopenia syndrome virus, cats, dogs, SFTS phlebovirus, veterinarians, zoonoses, viruses, Japan

## Abstract

In Japan, 2 cats that underwent surgery in a room where a sick dog had been euthanized became ill within 9 days of surgery. Severe fever with thrombocytopenia syndrome virus was detected in all 3 animals; nucleotide sequence identity was 100%. Suspected cause was an uncleaned pulse oximeter probe used for all patients.

Severe fever with thrombocytopenia syndrome (SFTS) is an emerging and mostly fatal tickborne zoonosis in eastern Asia. The causative agent is *Dabie bandavirus*, of the family Phenuiviridae and genus *Bandavirus*, and is generally known as SFTS virus (SFTSV). In Japan, SFTS-related mortality rates are reported to be 27% among humans and 62% among domestic cats (*1*,*2*). Although dogs can become infected with SFTSV, the mortality rate is unclear because infection of healthy dogs tends be subclinical (*3*). 

SFTSV is transmitted to humans and animals primarily through tick bites. However, nosocomial infection without a tick bite can occur via contact with blood and body fluids (*4*). Human-to-human transmission from an index patient to healthcare workers has been reported (*4*). Animal-to-human transmission from an index animal to veterinary personnel has also been reported (*5*,*6*). We report a nosocomial animal-to-animal transmission of SFTSV.

## The Cases

**Figure 1:**
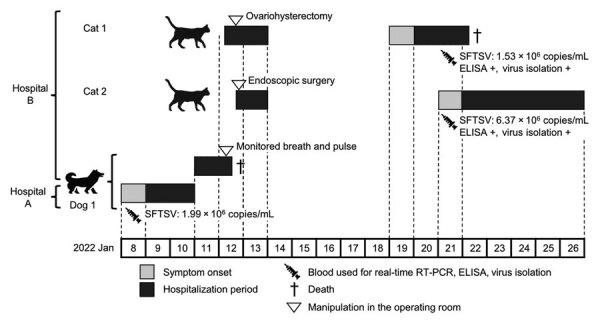
Timeline of dog-to-cat nosocomial transmission of SFTSV, Japan, 2022. Cat 1 was 7 months of age; cat 2 was 21 months of age; dog 1 was 13 years of age. RT-PCR, reverse transcription PCR; SFTSV, severe fever with thrombocytopenia syndrome virus; +, positive.

On January 8, 2022, a 13-year-old female dog (dog 1) with a high fever (39.9°C [reference range 38.0°C*–*39.0°C]) and anorexia was examined at animal hospital A (Table; Figure 1). The next day, dog 1 exhibited diarrhea and neurologic symptoms (unsteadiness and wandering). When the animal’s condition did not improve, on January 11, the dog was transferred to animal hospital B. On the basis of a high concentration of pancreas-specific lipase and pancreatic ultrasonography findings, veterinarians in animal hospital B diagnosed pancreatitis. Infectious disease was not suspected because the dog had no signs of a tick bite and had been vaccinated against most of the severe canine diseases in Japan. At 11:00 a.m. the next day, the dog was unresponsive to stimuli. The dog underwent tracheal intubation and mechanical ventilation, and a pulse oximeter probe was placed on the tongue. The dog did not respond to treatment and was euthanized and returned to the owner at approximately 3:00 p.m.

**Table:** Hematologic results and outcomes for 3 companion animals with severe fever with thrombocytopenia syndrome virus infection, Japan, 2022*

Characteristic	Reference ranges†			Dog 1			Cat 1		Cat 2
	Canine	Feline		Jan 8	Jan 12		Jan 21		Jan 21
Real-time RT-PCR, copies/mL	NA			1.99 × 10^6^‡	ND§		1.53 × 10^6^		6.37 × 10^6^
Virus isolation	NA			ND	ND		+		+
ELISA (absorbance at 405 nm)	(<0.04)§			ND	ND		+ (0.39)		+ (2.92)
Temperature, °C	38.0–39.0	38.0–39.0		39.9	ND		37.2¶		39.8
Leukocytes, × 10^2^ cells/mL	40–155	30–148		81.0	49.4		13.1		73.7
Hemoglobin, g/dL	12.1–20.3	9.3–15.9		14.4	11.4		15.1		35.1
Platelets, × 10^4^/mL	17–40	20–50		10.6	1.4		0.9		6.4
Total bilirubin, mg/dL	0.1–0.3	0.1–0.4		0.2	3.2		5.5		5.1
Alanine transaminase, U/L	12–118	10–100		157	189		566		148
Alkaline phosphatase, U/L	5–131	10–50		>1225	>1225		<10		16
Creatinine, mg/dL	0.5–1.6	0.6–2.4		0.8	5.1		1.3		1.5
Outcome				Euthanasia			Death		Recovery

*NA, not applicable; ND, not done; RT-PCR, reverse transcription PCR. †Standard canine and feline hematology parameters were selected according to (*7*). ‡Poorly preserved blood was used for real-time RT-PCR and virus isolation. §Cutoff value selected according to (*8*). ¶Temperature was 40.8°C on January 19.

On January 12, at approximately 10 a.m., a healthy 7-month-old female domestic cat (cat 1) was hospitalized at animal hospital B for ovariohysterectomy (Figure 1). At approximately 4:00 p.m., the ovariohysterectomy was performed under anesthesia on the same operating table and with the same ventilator used for dog 1. Cat 1 was discharged in healthy condition the next day.

Also on January 12, at approximately 6 p.m., a 21-month-old male domestic cat (cat 2) was urgently hospitalized at animal hospital B for ingestion of a foreign body. Cat 2 underwent endoscopic surgery under anesthesia in the same operating room and was discharged in healthy condition the next day. 

Cats 1 and 2 had no contact with dog 1 in the hospital. After surgery, the cats were kept in the same hospital room but in different cages and had no contact with each other. All 3 animals had different owners, and no contact before hospitalization was reported.

On January 19, a high fever (40.8°C [reference range 38.0°C*–*39.0°C]), vomiting, and inappetence developed in cat 1. Its condition worsened; on January 21, leukopenia and thrombocytopenia were confirmed (Table), and on January 22, the cat died. On January 21, high fever (39.8°C) with bilirubinuria developed in cat 2. Despite vomiting on January 22, cat 2 recovered by January 26. Both cats were kept indoors only, and neither had a history of a tick bite.

The director of animal hospital B suspected nosocomial infections because severe symptoms developed in the 2 cats that had undergone surgery on the same day. Serum samples from the cats were sent to the Center for Animal Disease Control, University of Miyazaki, where real-time reverse transcription PCR for SFTSV, feline calicivirus, and feline parvovirus was performed (*9*–*11*).

High copy numbers of SFTSV RNA were detected in both samples (cat 1 = 1.53 × 10^6^ copies/mL; cat 2 = 6.37 × 10^6^ copies/mL). Also confirmed by using double-antigen ELISA were IgG, IgM, or both against SFTSV nucleoprotein (absorbance at 405 nm) (cat 1 = 0.39; cat 2 = 2.92) (*8*). Blood collected from dog 1 on January 8 had been discarded in the medical waste box but was retrieved and sent to the Center for Animal Disease Control after results for the cats were confirmed. Although the blood had been kept at room temperature for >2 weeks, a high copy number of SFTSV RNA was detected (1.99 × 10^6^ copies/mL). ELISA was not performed because the blood was in poor condition. For veterinary personnel, body temperature and real-time reverse transcription PCR were monitored daily by the Oita City Public Health Center, but SFTSV infection was not detected.

**Figure 2:**
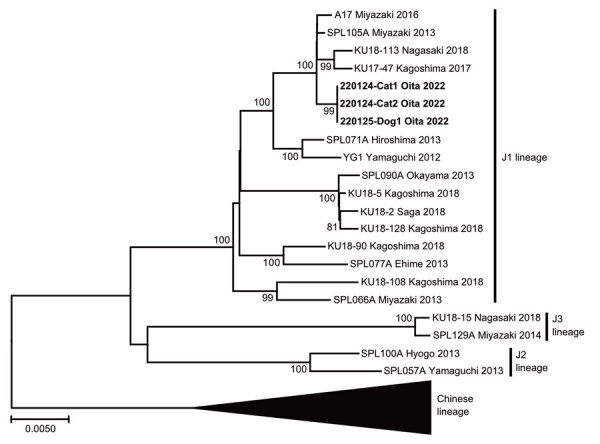
Phylogenetic analysis of severe fever with thrombocytopenia syndrome virus obtained from dog with index infection and 2 cats with nosocomial infection, Japan, 2022. The phylogenetic tree is shown for the viral genomic RNA of the medium segment. Boldface indicates viruses isolated from the animals in this study. Scale bar indicates nucleotide substitutions per site.

SFTSV isolation was performed by using serum from cats 1 and 2 and hemolyzed blood from dog 1. The virus isolation procedure has been previously described (*12*). SFTSV was isolated from both cats but not from the dog because of poor preservation of the dog sample. Next, the entire sequences of the SFTSV medium (M) segment from the animals were compared. The M segment encoding Gn and Gc glycoproteins is a more diverse segment than the small and large segments (*13*). Almost the entire sequence (SFTSV M segment, nt 9–3378) was successfully amplified and determined by using the reported primers (*13*) and submitted to the DNA Data Bank of Japan (accession no. LC705155-7). The virus sequences from the index dog and the 2 secondarily affected cats showed 100% homology (Figure 2). Furthermore, the sequences were most closely related (99.8%) to the SFTSV SPL105A Miyazaki 2013 strain (accession no. AB985315), which was obtained from a person with SFTS infection in an adjacent prefecture in 2013.

The operating room was a sanitary environment. The operating table was disinfected after each use; repeated use of contaminated instruments was prohibited; and all staff wore disposable gowns, masks, and gloves during operations. Although most medical instruments do not cause nosocomial infection, we determined that the pulse oximeter probe posed the highest risk for virus transmission between the dog and the cats because a disposable paper towel was placed between the probe and tongue, with saliva contaminating the probe, and the staff were unable to confirm whether the inner surface of the probe was wiped with hypochlorous acid between patients. A previous study detected high levels of viral RNA in the saliva of animals with SFTS (*14*,*15*). Because the same ventilator was used with the 3 animals reported here, aerosol transmission is another suspected source. Although the tracheal tubes and attached equipment were changed after each use, other parts (e.g., the breathing tube) were not changed and disinfected because infectious disease was not suspected.

## Conclusions

We report molecular evidence of nosocomial transmission of SFTSV among companion animals in an animal hospital in Japan. Veterinary personnel should be aware of the risk that this emerging zoonotic disease poses for their safety as well as the safety of patients and clients. To prevent nosocomial infections, veterinary staff should be educated about basic infection prevention and control practices in animal hospitals.
